# Toddler physical activity study: laboratory and community studies to evaluate accelerometer validity and correlates

**DOI:** 10.1186/s12889-016-3569-9

**Published:** 2016-09-06

**Authors:** Erin R. Hager, Candice E. Gormley, Laura W. Latta, Margarita S. Treuth, Laura E. Caulfield, Maureen M. Black

**Affiliations:** 1 Department of Pediatrics, University of Maryland School of Medicine, Baltimore, MD USA; 2 Department of Epidemiology and Public Health, University of Maryland School of Medicine, Baltimore, MD USA; 3 Center for Human Nutrition, Johns Hopkins Bloomberg School of Public Health, Baltimore, MD USA; 4 Family League of Baltimore, Baltimore, MD USA; 5 Department of Kinesiology, University of Maryland Eastern Shore, Princess Anne, MD USA

**Keywords:** Accelerometry, Toddler, Physical activity assessment, Very young children, Activity correlates, Low-income, Obesity prevention

## Abstract

**Background:**

Toddlerhood is an important age for physical activity (PA) promotion to prevent obesity and support a physically active lifestyle throughout childhood. Accurate assessment of PA is needed to determine trends/correlates of PA, time spent in sedentary, light, or moderate-vigorous PA (MVPA), and the effectiveness of PA promotion programs. Due to the limited availability of objective measures that have been validated and evaluated for feasibility in community studies, it is unclear which subgroups of toddlers are at the highest risk for inactivity. Using Actical ankle accelerometry, the objectives of this study are to develop valid thresholds, examine feasibility, and examine demographic/ anthropometric PA correlates of MVPA among toddlers from low-income families.

**Methods:**

Two studies were conducted with toddlers (12–36 months). *Laboratory Study* (*n* = 24)- Two Actical accelerometers were placed on the ankle. PA was observed using the Child Activity Rating Scale (CARS, prescribed activities). Analyses included device equivalence reliability (correlation: activity counts of two Acticals), criterion-related validity (correlation: activity counts and CARS ratings), and sensitivity/specificity for thresholds. *Community Study* (*n* = 277, low-income mother-toddler dyads recruited)- An Actical was worn on the ankle for > 7 days (goal >5, 24-h days). Height/weight was measured. Mothers reported demographics. Analyses included frequencies (feasibility) and stepwise multiple linear regression (sMLR).

**Results:**

*Laboratory Study-* Acticals demonstrated reliability (*r* = 0.980) and validity (*r* = 0.75). Thresholds demonstrated sensitivity (86 %) and specificity (88 %). *Community Study-* 86 % wore accelerometer, 69 % had valid data (mean = 5.2 days). Primary reasons for missing/invalid data: refusal (14 %) and wear-time ≤2 days (11 %). The MVPA threshold (>2200 cpm) yielded 54 min/day. In sMLR, MVPA was associated with age (older > younger, β = 32.8, *p* < 0.001), gender (boys > girls, β = −11.21, *p* = 0.032), maternal MVPA (β = 0.44, *p* = 0.002) and recruitment location (suburban > urban, β = 19.6, *p* < 0.001), or race (non-Black > Black, β = 18.5, *p* = 0.001). No association with toddler weight status.

**Conclusions:**

Ankle accelerometry is a valid, reliable, and feasible method of assessing PA in community studies of toddlers from low-income families. Sub-populations of toddlers may be at increased risk for inactivity, including toddlers that are younger, female, Black, those with less active mothers, and those living in an urban location.

## Background

Pediatric obesity is a serious public health problem, beginning early in life. Being overweight/obese in toddlerhood is associated with increased risk for overweight/obesity in Kindergarten [1], and excess weight gain before age 5 is maintained through adolescence [2, 3], increasing risk for obesity-related co-morbidities later in life and giving rise to the recommendation that obesity prevention programs begin early in life [4, 5]. Effective pediatric obesity prevention strategies should include a focus on physical activity (PA) behaviors [6, 7]. In addition to obesity prevention, PA is important in toddlerhood for the development of motor skills, bones/muscles, social skills, and cognitive growth/development [8].

The limited PA research on young children has focused on preschoolers (age 3–5 years), with relatively few studies focusing on toddlers (ages 12–36 months), despite the fact that active play contributes to toddler cognitive, physical, social, and emotional wellbeing among [9]. International PA guidelines have been developed for toddlers which recommend 180 min/day of total PA (light, moderate, and vigorous) [10–12], progressing toward 60 min of moderate-vigorous PA (MVPA) by age 5 [11]. These guidelines are based on limited empirical data, and studies are needed that objectively evaluate PA among toddlers in community settings in order to provide either support for existing guidelines or evidence for the need for updated guidelines.

Accurate assessment of PA is necessary to examine adherence to guidelines, PA trends and correlates, and to evaluate the effectiveness of PA promotion programs. Accelerometry provides a non-invasive method for objectively assessing PA and avoids proxy-report biases [13], which is important when working with young children for whom proxy-report would be necessary. To date, accelerometer validation studies have focused primarily on preschool-age children, [14–19] with limited methodological studies on toddlers [20]. Four studies used Actigraph accelerometry in controlled settings to determine validity and establish MVPA thresholds for toddlers using the hip or wrist placement [21–24]. Although hip placement provides more valid and reliable estimates of activity energy expenditure compared to ankle or wrist placement [25–27], hip placement has raised concerns about data volume and integrity in community studies [28–32]. Ankle placement has not been extensively studied in laboratory or home/community settings, yet may overcome limitations of hip and wrist placements through continuous, 24-h data collection (without periodic removal, common in hip placement, which often involves removal during sleep, bathing, and swimming [33]) and the ability to capture locomotion (difficult with wrist placement).

Due to the limited number of community studies of PA in toddlerhood, little is known about factors associated with PA in this population. Age is likely to be a primary correlate of toddler PA, given that during the transition from infancy to toddlerhood, children develop increasingly sophisticated gross motor skills, including walking and running. Later in toddlerhood and in the preschool years, children’s attentional and cognitive skills increase, leading to a decline in gross motor play and an increase in exploratory and problem-solving play (e.g. puzzles, imaginary play) [34]. Among preschoolers, accelerometry studies have identified demographic correlates of PA including male gender [35–39] and Black race (versus White) [36]. Additionally healthy weight preschoolers (versus overweight/obese) have been shown to engage in more PA [36, 37, 39, 40], PA associations between parents and their preschool-aged children are inconsistent [41, 42], and the parent-toddler PA association is unknown. Little is known about neighborhood influences on young children’s PA; a recent review found that school-aged suburban children were more active than urban children [43]. Studies are needed that objectively examine toddler PA in home/community environments to identify factors associated with PA.

The Toddler PA Study (TPAS) combines laboratory and community components to examine three objectives. The first is to determine the device-equivalence reliability/criterion-related validity and develop threshold counts [sedentary, light, and moderate-vigorous PA (MVPA)] for Actical ankle accelerometry among toddlers. The second is to examine the feasibility of ankle accelerometry in a community study of toddlers from low-income families. The final objective is to test the hypothesis that five demographic and anthropometric correlates documented previously among preschoolers are related to greater time in objectively measured MVPA among toddlers from low-income families: older age (versus younger), male (versus female), healthy weight (versus obese), greater maternal time spent in MVPA (versus less), suburban location (versus urban) and Black race (versus non-Black).

## Methods

### Laboratory study

#### Recruitment

Toddlers were recruited through mothers groups, flyers at daycare centers, and word of mouth to participate in a 1-day PA study. Eligibility included age 12–36 months, walks independently, no health problems that interfere with PA, and parent able to read/understand English. Procedures were approved by the University Institutional Review Board (IRB). Parents provided written informed consent on behalf of their toddler and completed a demographic questionnaire.

#### Accelerometry

Two Actical accelerometers (Philips Respironics, Minimitter, Bend, OR) were strung side-by-side on one hospital bracelet and fastened superior to the lateral malleolus of the non-dominant ankle (the left ankle was chosen if the parent indicated that the toddler had not demonstrated dominance), per the manufacturer's instruction. One Actical was randomly assigned as primary. Accelerometer counts were collected in 30-s intervals.

#### Child Activity Rating Scale (CARS)

The CARS [44] was used to assess real-time toddler activity. Although the CARS was originally validated among 3–4 year-old children, it has been used successfully with toddlers [21]. Intervals of 30-s were observed.

After attending a 1.5-h training, research assistants independently coded seven videos, 3–6.5 min in length, of toddlers engaging in activities (without pausing). Percent agreement and Kappa values were calculated when compared to a gold standard. Reliable raters (Kappa ≥0.75, Spearman correlation ≥0.8, % agreement >90 %) were selected.

#### Protocol

Protocol activities were chosen through a two-stage process. First, a survey of 13 toddler-typical activities was administered to parents of toddlers and health professionals (*n* = 10). Respondents indicated the intensity of each activity using the CARS intensity ratings. Nine survey activities, ranging in intensity, were selected for pilot-testing. The final protocol included six toddler-typical activities that represented a range of intensities which were chosen based on feasibility in the lab setting and ability to be maintained for approximately 6 min (the target observation time was 5 min, we extended this time by one minute in the protocol to allow for truncating when aligning data): (1) watching TV (sitting), (2) listening to a book (sitting), (3) table games, puzzles and play-doh (standing), (4) imaginary play, kitchen set and train table (walking), (5) ball games (running), and (6) chase/tag (running). One research assistant interacted with each toddler to maintain engagement and activity at a consistent CARS level for 6 min. A second research assistant rated the toddler’s activity using the CARS scale.

#### Data reduction and analysis

Actical software (version 2.12) was used to download accelerometer data. CARS data were aligned with accelerometer data (activity counts/30-s interval from the two Actical accelerometers) by participant and activity, removing times between activities when CARS data were not gathered. Data were reduced to ensure that only the time periods when toddlers were fully engaged in activities were retained by truncating the first and last 30 s of each activity (retaining ~5 min/activity).

Statistical analyses were completed using SPSS version 20.0. Device-equivalence reliability was determined using the intraclass correlation (ICC) between activity counts from the two Acticals worn concurrently. Criterion-related validity was determined using Spearman correlations between activity counts from the primary Actical and CARS values for the 30-s intervals.

Using the clean dataset (aligned activity counts/ CARS ratings for six activities), each 30-s interval was designated with an activity intensity threshold based on CARS rating [18]: Sedentary = CARS 1.0; Light = CARS 1.1–3.0; MVPA = CARS 3.1–5.0. The distribution of the means and standard deviations of the activity counts by intensity were examined graphically to identify clusters for each activity level [45]. Three researchers with accelerometry experience agreed upon thresholds for sedentary/light and light/MVPA to be tested. Thresholds were applied to the dataset, and sensitivity and specificity were calculated for each intensity level.

### Community study

#### Recruitment

Biological mothers and their toddler-age children (age 12–32 months, born at term, birth weight >2500 g, walks independently) were eligible. Subjects were recruited from two sites: a Special Supplemental Nutrition Program for Women, Infants, and Children (WIC) clinic in a suburban mid-Atlantic county and an inner-city pediatric clinic serving low-income families, both of which serve families living in the surrounding communities. Mothers provided written informed consent and completed self-administered, computer-based questionnaires using voice-generating software. This study was approved by University and State Department of Health Institutional Review Boards. Evaluations were conducted during two visits, separated by one week. Mothers reported on their toddlers’ birth date, gender, and race/ethnicity and on their own birth date, marital status (categorical responses; dichotomized as married versus not married), education (categorical responses for highest degree; dichotomized based on some high school versus high school diploma/equivalent or higher), and number of household members/annual household income (used to calculate a poverty ratio based on household income and number of dependents using U.S. Census Bureau 2009 thresholds [46]).

#### Anthropometrics

Mothers undressed their toddler to a clean diaper/underpants. Weight (kg) was measured in triplicate using a TANITA 1584 Baby Scale (TANITA, Tokyo, Japan). Recumbent length (cm) was measured in triplicate using a Shorr Measuring Board (Shorr Productions, Olney, Maryland). Gender-specific weight-for-length percentiles were calculated using CDC growth charts [47]. Obesity was defined as ≥ 95th percentile weight-for-length. Maternal height (cm) was measured in triplicate using a Shorr Measuring Board, and body weight (kg) was measured in duplicate (TANITA 300GS, Tanita Corp., Tokyo, Japan). If two measures differed by more than a centimeter (length or height) or differed at all (weight) then subsequent measures were taken, with the final measures averaged. BMI was calculated as weight (kg)/height (m)^2^, with overweight and obesity defined as BMI 25–29.9 kg/m^2^ and BMI ≥ 30 kg/m^2^, respectively.

#### Accelerometry

Both the toddlers and their mothers wore an Actical accelerometer, strung on a hospital bracelet and fastened superior to the lateral malleolus of the non-dominant or left ankle, per the manufacturer's instruction (next to the skin, under socks; once latched, the band cannot be removed unless cut off). The accelerometers were to be worn for at least seven consecutive days without removal as toddlers and mothers engaged in their routine activities. The Actical is small, light, and waterproof, and can be worn during sleep, play, bathing or swimming. For both mothers and toddlers, activity counts were collected in 1-min intervals to provide direct comparison for mothers and toddlers. During the second visit, the Actical band was removed.

#### Data reduction and analysis

Accelerometer data for both mothers and toddlers were reduced using Actical software (version 2.12). Days with <80 cpm were treated as incomplete and removed. Only days with complete data (i.e. 24-h period) were included, therefore the first and last day of wear time were truncated. If the accelerometer was removed on the second day (≤2 days of wear time), then the data were considered invalid and excluded. Data were considered valid if at least one 24-h period day (12:00 am–11:59 pm) was recorded. For participants with >7 days, data were truncated after the 7th day. Thresholds were applied to the clean dataset to determine time spent in sedentary (including sleep), light, and MVPA for mothers [45] and toddlers (newly developed from the laboratory study).

Feasibility of toddler ankle accelerometry was determined by the proportion of toddlers with valid accelerometer data. Reasons for incomplete data were recorded. *T*-test and Chi-square analyses were employed to compare how the sample differed on demographic variables by the presence/absence of valid accelerometry data. The thresholds from the Laboratory Study (based on 30-s intervals) were proportionately converted into 1-min intervals (multiplied by 2) [45]. Time in each PA level was calculated for toddlers using thresholds from the Laboratory Study and for mothers from a previously validated threshold [45]. Demographic and anthropometric correlates of PA were examined using Spearman correlations, independent t-tests, and chi-square analyses. Variables associated with MVPA were tested for collinearity with the Variance Inflation Factor (VIF). Skewness of minutes in MVPA was examined. Step-wise multiple linear regression was used to identify variables associated with toddler MVPA in a single model.

## Results

### Laboratory study

Twenty-four toddlers participated (mean age: 24.5 months, range 14.7–35.5), 45.8 % were <24 months, and 58.3 % were male. All toddlers completed the six activities, with a mean duration of valid data ranging from 5 min 15 s to 6 min 21 s. The selected activities increased in activity counts and CARS values as the motion changed, yielding a wide range of values (Table 1).

**Table 1:** Means ± standard deviations for simultaneous activity counts and activity ratings during prescribed activities (30 s intervals, *n* = 24)

	Motion	Accelerometer activity counts (Actical, ankle)	Observed activity ratings (CARS)
Watching TV	Sitting	152 ± 175	1.6 ± 0.5
Listening to a Book	Sitting	111 ± 157	1.7 ± 0.3
Table games	Standing/Slow Walking	183 ± 251	2.1 ± 0.2
Imaginary play	Standing/Slow Walking	301 ± 183	2.3 ± 0.2
Ball games	Running	1029 ± 414	2.8 ± 0.2
Chase/Tag	Running	1932 ± 888	3.2 ± 0.4

The device equivalence reliability of the Actical (ICC between two accelerometers on the same ankle concurrently) was 0.980. Criterion-related validity (correlation between primary Actical accelerometer and CARS ratings) was 0.749.

**Fig. 1:**
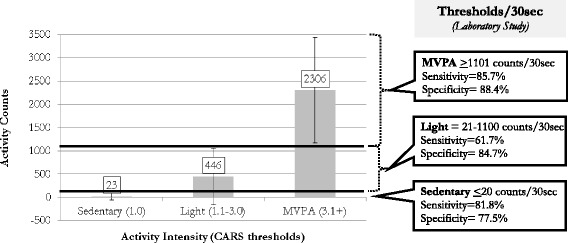
Activity Counts by Intensity (Laboratory Study). The bar graph represents the means (± standard deviations) of accelerometer activity counts by intensity. Bolded lines indicate thresholds designated for sedentary/light and light/MVPA with the sensitivity and specificity of thresholds presented in the right panel

Activity counts (mean ± standard deviation [SD]) were plotted by CARS intensity [18], merging data from all 6 activities (Fig. 1). As shown in Fig. 1, for the CARS threshold of 1.0 (sedentary) the mean activity count/30 s was 23. The threshold distinguishing sedentary activity from light activity was set at the approximate mean sedentary activity count (20 counts/30 s). The 20 counts/s threshold for sedentary activity had a sensitivity of 81.8 % and a specificity of 77.5 %. The threshold distinguishing light activity from MVPA was set at 1100 counts/30 s, based on the mean + SD for light activity (1055 counts/30 s) and mean–SD for MVPA (1171 counts/ 30 s, Fig. 1). Considering the threshold of >20 counts/30 s and <1100 counts/30 s for light activity, sensitivity was 61.7 % and specificity was 84.7 %. The 1100 counts/30 s threshold had a sensitivity of 85.7 % and a specificity of 88.4 % meaning that 85.7 % of MVPA intervals and 88.4 % of the non-MVPA intervals were accurately categorized.

### Community study

A total of 277 low-income mother-toddler dyads were recruited to participate and most mothers (*n* = 239, 86.3 %) agreed to allow their toddler to wear the accelerometer. Using a criterion of one full 24-h day of data, 191 had valid data. Mean number of days of valid data was 5.2 (range 1–7 days). Reasons for missing data (Table 2) include participant noncompliance [refusal to participate (13.7 %)] and wearing the accelerometer for ≤2 days (10.8 %).

**Table 2:** Reasons for missing toddler ankle accelerometer data (*n* = 277)

Category	Specific reasons	*N* (%)
Valid data	--	191 (69.0 %)
Refusal	Refused to participate in physical activity assessment	38 (13.7 %)
Participant noncompliance	Lost or did not return the accelerometer	3 (1.1 %)
	Wore accelerometer for less than two days^a^	30 (10.8 %)
Technical problems	Staff training (i.e.: improper programming and documentation)	11 (3.9 %)
	Device malfunction	4 (1.5 %)

^a^because the first and last days are truncated, if the participant wore the accelerometer for two or fewer days the data were not used

No differences between toddlers with/without data were observed by age, gender, race, or body size (weight-for-length), nor by maternal age, education, body size (BMI), number of household children, or poverty ratio. Toddlers with valid data were more likely to be suburban (76.4 %) versus urban (64.1 %, *χ*^2^ = 4.7, *p* = 0.031), and to have mothers with valid data (92.1 % versus 7.9 %; *χ*^2^ = 58.3, *p* < 0.001).

Demographic characteristics of this sample (*n* = 191, Table 3) include toddler age from 12.0 to 31.9 months, with most (73.3 %) <24 months, over half (53.9 %) were male, 67.5 % were Black (survey response: “African American or Black”), and 11.5 % were obese. Mothers were, on average, 26.8 years, the majority were unmarried (71.7 %), most had a high school diploma or equivalent (83.8 %), and the majority were overweight/obese (71.7 %). Mothers engaged in <30 min/day in MVPA (26.5 min/day). Most families lived at or below the poverty threshold (66.0 %), over half were recruited from the urban site (56.0 %), and average family size was 2.5 children.

**Table 3:** Toddler and maternal characteristics and correlates of toddler minutes/day in MVPA (*n* = 191)

	Mean ± SD/(range) or %	Mean minutes in MVPA	Association with minutes in MVPA	
			T (p)/F (p)	*r* (p)
Toddler characteristics				
Physical activity (minutes/day)				
Sedentary (including sleep)	803.2 ± 100.6	--	--	--
Light	582.4 ± 87.5	--	--	--
MVPA	54.1 ± 40.4	--	--	--
Days of valid accelerometer data				
Number of days	5.3 (1–7)	--	--	−0.07 (0.352)
1–4 days	19.4 %	56.4	0.31 (0.761)	
5–7 days	80.6 %	53.5		
Age				
Months	20.1 (12.0–31.9)	--	--	0.43 (<0.001)
≤ 24 m	73.3 %	43.4	6.72 (<0.001)	--
> 24 m	26.7 %	83.4		
Gender				
Male	53.9 %	60.1	2.24 (0.027)	--
Female	46.1 %	47.0		
Race				
Black	67.5 %	50.3	1.88 (0.062)	--
Other	32.5 %	61.9		
Body size				
Weight-for-length %tile	50.7 (0.1–99.99)	--	--	−0.09 (0.221)
Healthy weight (<94.9th %tile)	88.5 %	55.8	2.30 (0.027)	--
Obese (≥95th %tile)	11.5 %	40.9		
Maternal characteristics				
Age				
Years	26.8 (18–44)	--	--	0.07 (0.344)
Marital status		--		
Married	28.3 %	56.1	0.42 (0.672)	--
Not Married	71.7 %	53.3		
Education				
Some high school	16.2 %	52.9	0.19 (0.854)	--
≥ High school diploma/equivalent	83.8 %	54.3		
Physical activity				
MVPA (minutes/day)	26.5 (0–120)		--	0.18 (0.017)
Body size				
BMI (kg/m^2^)	31.3 (16.5–68.3)	--	--	−0.11 (0.119)
Normal weight (<25 kg/m^2^)	28.3 %	56.9	1.78 (0.172)	--
Overweight (25–29.9 kg/m^2^)	20.4 %	62.7		
Obese (≥30 kg/m^2^)	58.3 %	49.1		
Poverty				
Above	31.4 %	59.0	1.16 (0.246)	--
At/Below	66.0 %	51.6		
Recruitment location				
Urban	56.0 %	48.9	2.02 (0.045)	--
Suburban	44.0 %	60.7		
Household composition				
Number of children	2.5 (1–6)	--	--	−0.09 (0.216)

Accelerometry thresholds applied in the Community Study were: 0–40 (Sedentary), 41–2200 (Light), ≥2201 (MVPA) counts/min, which, when applied, showed that toddlers were engaging in an average of 803 min/day in sedentary activity (including sleep), 582 min/day in light activity, and 54 min/day in MVPA (24 h periods, Table 3). To calculate total PA, light and MVPA are included together, yielding an average of 637 min/day. On average, toddlers had 5 days of valid data (ranging from 1 to 7). Few toddlers (5.8 %) had only one day of valid data. Associations between number of days of valid data and time in MVPA, examined continuously or categorically, were not observed (Table 3).

Correlates of time spent in MVPA (Table 3) show that older versus younger toddlers were more active (*p* < 0.001, toddlers aged 24–32 months engaged in nearly twice the amount of activity compared to toddlers 12–24 months of age), male toddlers were more active than female (~13 additional minutes MVPA/day, *p* = 0.027), and healthy weight toddlers were more active than obese (~15 additional minutes MVPA/day, *p* = 0.027). Non-Black toddlers were marginally more active than Black (~11 additional minutes MVPA/day, *p* = 0.062). Maternal time in MVPA was positively associated with toddler MVPA (*r* = 0.18, *p* = 0.017) and suburban toddlers were more active than urban (~12 additional minutes in MVPA/day, *p* = 0.045).

In step-wise multiple linear regression models, minutes/day in MVPA was marginally skewed (Skewness = 1.040), and not transformed for interpretation. Variables that were significantly or marginally associated with minutes in MVPA in the bivariate analyses included toddler age, gender, obese status, race, maternal PA, and recruitment location. The majority (90.7 %) of the urban sample was Black compared to 38.1 % of the suburban sample (*χ*2 = 59.3, *p* < 0.001). The VIF indicated marginal collinearity (1.5, Tolerance = 0.68), however given prevalence differences in race by location, two models were run, one including race and one including recruitment location.

Both stepwise regression models excluded obese status; other variables were retained (Table 4). In both, age was included first, followed by recruitment location or race, then maternal PA, then gender. Each variable was significantly associated with toddler MVPA, in the same direction as the bivariate associations. The final models, with all included variables, had *R*^2^ values of 0.28–0.29, indicating nearly 30 % of the variance in MVPA explained.

**Table 4:** Stepwise multiple linear regression models examining associations with minutes in MVPA

	Model #1				Model #2			
	B	Std. Error	*p*	*R* ^2^	B	Std. Error	*p*	*R* ^2^
1 Age^a^	38.6	6.1	<0.001	0.19	38.6	6.1	<0.001	0.19
2 Age^a^	40.7	5.9	<0.001	0.23	40.4	5.9	<0.001	0.23
Recruitment Location^b^	16.5	5.3	0.002		--	--	--	
Race^b^	--	--	--		17.2	5.6	0.002	
3 Age^a^	39.2	5.8	<0.001	0.27	39.0	5.9	<0.001	0.26
Recruitment Location^b^	18.4	5.2	0.001		--	--	--	
Race^b^	--	--	--		17.8	5.5	0.001	
Maternal PA (min/day in MVPA)	0.41	0.1	0.004		0.36	0.14	0.011	
4 Age^a^	38.2	5.8	<0.001	0.29	38.0	5.5	<0.001	0.28
Recruitment Location^b^	19.6	5.2	<0.001		--	--	--	
Race^b^	--	--	--		18.5	5.5	0.001	
Maternal PA (min/day in MVPA)	0.44	0.1	0.002		0.39	0.14	0.006	
Gender^c^	−11.2	5.2	0.032		−10.3	5.2	0.048	

^a^0 = <24 months, 1= > 24 months

^b^0 = urban, 1 = suburban; 0 = black, 1 = other

^c^0 = male, 1 = female

## Discussion

This paper describes two studies that together validate ankle accelerometry among toddlers and describe low-income toddler PA. As obesity prevention efforts focus on younger children for whom little is known about PA, validated objective PA measures and descriptive/correlational studies are needed.

The Laboratory Study showed that ankle accelerometry placement is valid for measuring variations in PA compared to direct observation and can reliably record movement. Second, sensitive and specific thresholds for sedentary, light, and MVPA were created, relying on CARS as a criterion method [18, 19, 21]. Varying CARS MVPA thresholds have been used in accelerometer validation studies, including ≥3.0 [21], ≥3.1 [18], and ≥4.0 [19, 24]. We used the ≥3.1 threshold for MVPA because a CARS score >3.0 means that a portion of the interval included activity greater than light. By comparing activity counts to simultaneously CARS-generated observational activity intensity data, we demonstrated criterion-related validity of the toddler Actical ankle accelerometry while also generating intensity thresholds. This study adds to the new but growing literature on methods for objectively assessing PA among toddler-aged children [21–24] by providing support for an alternative accelerometer placement (ankle) and monitor (Actical).

The Community Study yielded three findings. First, ankle accelerometry is feasible for measuring PA among toddlers from low-income families, demonstrated by the high acceptance rate (86.3 %) and proportion of valid data (69.0 %). Most missing data was due to participant non-compliance, including device removal within 48 h. Several strategies were used to reduce refusals and maintain compliance: (1) incentive for accelerometer return, (2) accelerometer band decorated with stickers, and (3) mothers and toddlers wore ankle accelerometers concurrently. Future studies should explore mechanisms for reducing refusals and enhancing compliance among this young age group. Much of the toddler PA research to date has involved only laboratory accelerometer validation studies [21, 23, 24] with little research conducted in community settings. A study by Van Cauwenberghe et al. examined the feasibility of hip accelerometry among a small sample (*n* = 47) of toddlers, 12–30 months of age, over ~6 days and found this method to be feasible [22]. For this study, a minimum of 7.5 h/day of wear-time was considered valid (average wear-time was 9.4 h/day) [22]. The current study adds to the literature by providing evidence for a valid and feasible method of obtaining 24-h accelerometry data among toddlers in community settings.

Second, toddlers were engaging in an average of 54.1 min of MVPA/day. Recent international PA guidelines set recommendations for total PA for toddlers (light and MVPA together) at 180 min/day, with a progression toward 60 min of MVPA/day by age 5 [10–12]. In this study, toddlers from low-income families, on average, were not reaching 60 min of MVPA/day, however older toddlers, ages 24–32 months, exceeded 60 min/day. Future studies should examine the progression of time spent in MVPA from toddlerhood through the preschool years.

Third, activity was associated with older age, male gender, non-Black race, and suburban location. Based on motor skill development, we hypothesized that older toddlers would be more active than younger, which was supported. Older toddlers engaged in nearly twice the amount of MVPA compared to younger toddlers. We expected age to be a primary correlate of toddler PA, given the developmental changes occurring during this period. The large difference observed in this study warrants further examination, perhaps over a broader age range (i.e.: 0–5) or by following young toddlers longitudinally. We hypothesized that male toddlers would be more active than female, based on studies of preschool-aged children, which was supported, with male toddlers engaging in nearly 13 more minutes per day of MVPA. A gender difference in PA observed prior to the preschool years is an important finding. Factors that led to this gender difference and the long-term impact of an early difference in PA by gender should be examined further. Black toddlers were less active than White, however, most Black toddlers were recruited from an urban location and most White toddlers were recruited from a suburban location. Toddlers recruited from the suburban location were more active (~16 additional minutes/day in MVPA) compared to urban toddlers in adjusted models. There is little research examining neighborhood environment and PA among young children, prior to school-age. Base on the findings from this study, additional research is needed to understand how the neighborhood environment relates to toddlers’ PA, and the relation between race/residence and PA among very young children. Maternal PA was associated with toddler PA, as hypothesized. Longitudinal studies should examine the direction of this relation, specifically whether being an active mother leads to having active toddlers or if having an active toddler increases maternal PA or if the relationship is transactional. Finally, this study demonstrated that, when adjusting for covariates, there was no difference in PA by toddler weight status.

Recently updated PA guidelines from three countries state that toddlers should engage in 180 min of total PA each day (including light and MVPA) [10–12]. The TPAS found that, using objective measures in a sample of toddlers from low-income families in the U.S., total PA averaged 637 min/day (over 3.5 times the amount stated in the guidelines). Few community-based studies of toddlers using objectively measured PA have been conducted to inform guidelines. Further, these guidelines describe total PA in toddlerhood as including light activities such as “standing up, moving around” [12] and “moving around the home” [11]. Findings from this TPAS suggest that the current PA guidelines may significantly underestimate the amount of time toddlers are already engaging in total PA. Also, a recent review of 14 studies including over 20,000 children age 4–18 years found that more time in MVPA was associated with cardiometabolic risk factors, regardless of sedentary time [48], which suggests that MVPA in toddlerhood alone should be considered in future studies. Additional objective community-based studies of PA in toddlerhood are warranted to inform/generate evidence-based toddler PA guidelines.

It has been recommended that PA of very young children be measured in small intervals (i.e.: 15-s) to record short bursts of movement [20]. We chose to record data in 30-s intervals for the laboratory study and 1-min intervals in the community study for several reasons. First, the community study was designed such that the mother and toddler PA data were collected together, using the same interval of time (1-min), for comparison. Second, in a prior study we were able to aggregate thresholds based on Actical interval length up for longer durations of time [45], therefore we designed this laboratory study to capture Actical and CARS data in smaller increments (30-s) with the intention of aggregating up to 1-min for application in the community study. Finally, the standard protocol for the laboratory criterion measure (CARS) prescribes 1-min intervals [44], which we were able to reduce to 30-s and maintain data quality.

There are several strengths and limitations associated with these studies. Strengths include ankle placement offering continuous, 24-h data collection, with minimal participant burden (i.e., no need to remove the device during bathing/sleep), while also capturing movements that involve translocation; however a limitation of this method is the inability to differentiate sedentary time from sleep, which may be particularly pertinent during toddlerhood. Using accelerometry to determine MVPA time and PA correlates is a strength, given that accelerometry avoids biases of proxy-reporting (necessary for young children). The Community study allowed for a unique examination of an array of PA correlates in a sample of toddlers from low-income families, including dual accelerometer comparisons of PA among mothers and their toddler-aged children. The choice to collect toddler data in one-minute epochs, for comparison with maternal accelerometer data, is also a limitation as current best practice is to measure PA of very young children in 15 s epochs. The current study, as conducted, is able to provide valuable information about toddler PA (including in relation to maternal PA), albeit the recorded intervals of time are longer than recommended. The MVPA threshold applied to the mothers’ accelerometer data was derived from a study of adolescent girls [45] and not developed specifically for adult women; however no valid threshold is available specifically for Actical ankle accelerometry among adult women (when applying thresholds built into the manufacture's software time spent in MVPA is dramatically overestimated for the ankle placement [45]). The Community Study sample was exclusively low-income, a population at risk for obesity and inactivity; however, the homogeneity also reduces generalizability to other populations.

## Conclusions

This study demonstrated that Actical ankle accelerometry is a valid and feasible method of assessing PA in community studies of toddlers from low-income families. Sub-populations of toddlers may be at increased risk for inactivity, and interventions should consider these when determining whom to target and how to design PA promotion interventions involving toddlers.

## Abbreviations

BMI: Body mass index
CARS: Child Activity Rating Scale
MVPA: Moderate to vigorous physical activity
PA: Physical activity
WIC: Special supplemental nutrition program for women, infants, and children
